# Sleeping Position

**Published:** 1877-04

**Authors:** 


**SLEEPING POSITION.**—The food passes from the stomach at the right side, hence its passage is facilitated by going to sleep on the right side. Water and other fluids flow equably on a level, and it requires less power to propel them on a level, than upwards. The heart propels the blood to every part of the body at each successive beat, and it is easy to see that if the body is in a horizontal position the blood will be sent to the various parts of the system with greater ease, with less expenditure of power, and more perfectly than could possibly be done if one portion of the body were elevated above a horizontal line. On the other hand, if one portion of the body is too low, the blood does not return as readily as it is carried thither; hence, there is an accumulation and distention, and pain soon follows. If a person goes to sleep with the head but a very little lower than the body, he will either soon waken up, or will die with apoplexy before the morning, simply because the blood could not get back from the brain as fast as it was carried to it. If a person lays himself down on a level floor for sleep, a portion of the head, at least, is lower than the heart, and discomfort is soon induced; hence, very properly, the world over, the head is elevated during sleep. The savage uses a log of wood or a bunch of leaves; the civilized a pillow; and if this pillow is too thick, raising the head too high, there is not blood enough carried to the brain, and as the brain is nourished, renewed, and invigorated by the nutriment it receives from the blood during sleep, it is not fed sufficiently, and the result is unquiet sleep during the night, and a waking up in weariness, without refreshment, to be followed by a day of drowsiness, discomfort, and general inactivity of both mind and body. The healthful mean is a pillow, which by the pressure of the head keeps it about four inches above the level of the bed or mattress; nor should the pillow be so soft as to allow the head to be buried in it, and excite perspiration, endangering ear-ache or cold in the head, on turning over. The pillow should be hard enough to prevent the head sinking more than about three inches.

One of the most valuable books a person can possess is a good encyclopedia. There is no better encyclopedia published than Zell's. See advertisement in this JOURNAL.

